# Art and Psychological Well-Being: Linking the Brain to the Aesthetic Emotion

**DOI:** 10.3389/fpsyg.2019.00739

**Published:** 2019-04-04

**Authors:** Stefano Mastandrea, Sabrina Fagioli, Valeria Biasi

**Affiliations:** ^1^ Department of Education, Experimental Psychology Laboratory, Roma Tre University, Rome, Italy; ^2^ Neuroimaging Laboratory, IRCCS Santa Lucia Foundation, Rome, Italy

**Keywords:** aesthetic emotion, art museum, art-based learning, neuroaesthetics, well-being, emotion regulation, aesthetic appraisal

## Abstract

Empirical studies suggest that art improves health and well-being among individuals. However, how aesthetic appreciation affects our cognitive and emotional states to promote physical and psychological well-being is still unclear. In this review, we consider the idea that the positive emotional output elicited from the aesthetic experience affects mood, and indirectly promotes health and well-being. First, we examine evidence that arts promoting well-being involve art museums, healthcare settings, and education. Second, we review some neuroimaging studies addressing aesthetic experience and emotional processing. In particular, we leveraged advances in neuroaesthetics to explore different hypotheses about the determinants of aesthetic pleasure during art reception, in the attempt to clarify how experiencing art promotes well-being. Finally, we propose research on aesthetic experience and psychophysiological measures of stress, with the goal of promoting a focused use of art as a tool for improving well-being and health.

## Introduction

Aesthetic experience concerns the appreciation of aesthetic objects and the resulting pleasure. Such pleasure is not derived from the utilitarian properties of the objects but linked to the intrinsic qualities of the aesthetic objects themselves. Hence, the aesthetic pleasure is disinterested (Kant, 1790). Aesthetic experiences can arise from the appreciation of human artifacts, such as artworks (e.g., poetry, sculpture, music, visual arts, etc.) or aesthetic natural objects like sunsets or mountain vista. In this review, we refer to aesthetic experiences associated with the appreciation of artworks, particularly visual arts.

Aesthetic experiences are offered by multiple contexts, (e.g., museums, galleries, churches, etc.). Several psychological perspectives considered aesthetic experience as a rewarding process and suggested a link between aesthetic experience and pleasure (Berlyne, 1974; Leder et al., 2004; Silvia, 2005). Recent studies suggest the arts can promote health and psychological well-being and offer a therapeutic tool for many, e.g., adolescents, elderly, and vulnerable individuals (Daykin et al., 2008; Todd et al., 2017; Thomson et al., 2018). Aesthetic experience has been associated with mindfulness meditation, as it leads to enhancing the capability of perceptually engaging with an object (Harrison and Clark, 2016). However, how aesthetic experience affects cognitive and emotional states and promotes physical and psychological well-being is a matter of debate (Daykin et al., 2008). Several theoretical models have been proposed, suggesting alternating key roles for cognitive or emotional facets of the aesthetic experience. A common theme in the models is that the aesthetic evaluation of an artwork is the result of bottom-up stimulus properties and top-down cognitive appraisals (Leder et al., 2004; Chatterjee and Vartanian, 2016; Pelowski et al., 2017). The result affects mood, therefore promoting health and well-being (Kubovy, 1999; Sachs et al., 2015).

In this vein, neuroimaging studies highlighted that immediate emotional responses to artwork and low-intensity enduring changes in affective states (cf. Scherer, 2005, for the distinction of emotional response and affective state) are associated with recruitment of brain circuitry involved in emotion regulation, pleasure, and reward. Thus, for instance, images rated as beautiful elicit activity in reward-related areas, such as the medial orbitofrontal cortex, and are associated with higher reward value than those rated as ugly (Kawabata and Zeki, 2004). Moreover, the activation of an emotion processing network comprising the ventral and the dorsal striatum, the anterior cingulate and medial temporal areas has been associated with the transient mood changes in response to happy and sad classical music (Mitterschiffthaler et al., 2007).

Here, we review evidence showing that arts promote well-being across several domains, and discuss the neural underpinnings of aesthetic experience, emotional processing, pleasure, and reward. In particular, we assess the idea that a common physiological mechanism underlies aesthetic processing in multiple places for experiencing art. Implications for therapeutic and educational uses of art are discussed.

### Aesthetic Appreciation and Well-Being

Benefits associated with aesthetic processing have been demonstrated in different settings, ranging from reproductions of paintings shown in laboratories to real art contexts such as museums.

In the following sections, we present a review of the main research branches on art in which a beneficial effect on health has been shown.

#### Art in the Museum

Several studies show benefits of art museums as settings for therapy (Treadon et al., 2006; Chatterjee and Noble, 2013). These benefits include improvement of memory and lower stress levels, and amelioration of social inclusion. Populations studied include older individuals (Salom, 2011; Thomson et al., 2018), people with enduring mental health problems (Colbert et al., 2013), people with dementia (Morse and Chatterjee, 2018), and the socially isolated (Todd et al., 2017). Moreover, in a study with people with dementia and their caregivers, viewing traditional and contemporary galleries, both art sites promoted well-being, including positive social impact and cognitive enhancement (Camic et al., 2014).

Research has been conducted to identify the elements of the museum setting that facilitate the treatment goals, including psychological, social, environmental aspects (Salom, 2011; Camic and Chatterjee, 2013; Colbert et al., 2013; Morse and Chatterjee, 2018). Museum environment and artifacts offer an extraordinary aesthetic experience that allows the recollection of positive memories (Biasi and Carrus, 2016), and evidence suggests that these reminiscence activities can affect mood, self-worth, and a general sense of well-being in the elderly (Chiang et al., 2009; O’Rourke et al., 2011; Eekelaar et al., 2012). Museum and galleries, unlike hospitals and clinics, are non-stigmatizing settings. The art setting encourages self-reflection and group communication, facilitating the therapeutic process and thus making them ideal locations for health interventions (Camic and Chatterjee, 2013).

Using psychophysiological measures, studies find visits to art museums decrease stress, which could promote health and well-being (Clow and Fredhoi, 2006; Mastandrea et al., 2018). Clow and Fredhoi reported that levels of salivary cortisol and self-reported measure of stress in 28 healthy young individuals decreased significantly after a visit to the Guildhall Art Gallery of London (Clow and Fredhoi, 2006). Similarly, exposure to figurative art lowers systolic blood pressure (SBP), which could have relaxing effects (Mastandrea et al., 2018). Specifically, 64 healthy female participants were assigned to one of three different visits to the National Gallery of Modern Art in Rome: figurative art, modern art, and a control condition consisting of a visit to the museum office. Pre- and post-visit measures of blood pressure and heart rate were acquired, as indices of emotional states associated with the three visit conditions. Results revealed that only figurative art exposure decreased systolic blood pressure. Of interest, participants liked the two art styles equally well, and reduction in SBP was not correlated with liking. In fluency theory, processing ease increases positive emotional response to artwork (Reber et al., 2004). Accordingly, it may be thought that the reduction of levels of ambiguity that characterizes unambiguous figurative arts may have a relaxing effect on the physiological states. On the other hand, as participants in this study were not asked to judge the comprehensibility or hedonic values of artworks, it is not possible to draw firm conclusions about the restorative effects following exposure to figurative, but not abstract artworks in art museum.

#### Art and Education

Several studies have been conducted on the efficacy of art-based interventions in professional education, demonstrating a growing interest for this field, and posing challenging opportunities for the traditional learning methods that shape the current teaching practice (Richard, 2007; Leonard et al., 2018). Art-based pedagogy is focused on the integration of an art form (e.g., theatre, visual art-painting, music, etc.) with another subject matter, to enhance learning processes (Rieger and Chernomas, 2013).

In learning through art, the learner approaches a subject matter by creating art, responding to art, or performing artistic works not by studying art as a theoretical discipline (Rieger and Chernomas, 2013). This art-based learning (ABL) has been used successfully in healthcare education (Wikström, 2003; Rieger et al., 2016). For instance, using a work of art as a teaching method is effective in increasing students’ observational skills, empathy (i.e., abilities in empathizing with the patient and develop compassion), nonverbal communication, and interpersonal relationships, in comparison with traditional teaching programs (Wikström, 2011). Wikstrom (2000) and colleagues showed that an educational program based on visual art dialogue evoked emotional experiences increasing nurses’ empathy (Wikstrom, 2000). The students were asked to describe nursing care patterns in the painting “The Sickbed” from Lena Croqvist, after which they were asked strategic questions aimed at eliciting empathetic responses, such as “From a nursing care perspective, how do the characters feel?” A control group was asked to describe good nursing practice without the support of visual art or pictures. The visual art was more effective than the control for expressing aspects of nursing care and in increasing empathy scores (Wikström, 2001). These studies suggest that embedding visual art in healthcare education may increase understanding of emotional experience of chronic pain and suffering of the patients, thereby improving nursing care practices. A limitation of these studies is that control groups received only verbal instruction, that make it difficult to evaluate the specific contribution of art-specific visual support (i.e., visual portraits, artworks, etc.) from nonartistic visual support. On the other hand, correlational studies show that high aesthetic value of artistic movie sequences perceived by the student is associated significantly with learning enhancement (Bonaiuto et al., 2002).

One might wonder how the emotional experience elicited by the appreciation of diverse forms of art enables individuals to feel better and learn quickly and effectively, and whether the boosting effect of art on these different domains forms a basis of a common cognitive or affective mechanism. Here, we suggest that the processing of aesthetic artwork relies on the activity of reward-related brain areas, resulting in positive emotions and pleasure that, modulating affective state, increase the individual predisposition to cognitive activities such as learning.

### Linking the Brain to Aesthetic Experience

The studies reviewed so far demonstrated that the aesthetic value of artwork and their use in educational programs may affect psychological and physiological states, thus promoting well-being and enhancing learning. However, as we stated above, the mechanisms underlying the relationship between art and well-being are still unclear, probably due to the fact that the determinants of the aesthetic experience and its relationship with emotion processing and pleasure are still unresolved.

Here, we review some neuroimaging evidence detailing the neural underpinnings of the relationship between aesthetic experience and activation of emotional states in the beholder, to provide a more comprehensive understanding of the aesthetic experience and how it provokes aesthetic emotion and pleasure in the beholder. Moreover, we relate these findings to influential models of aesthetic processing.

From a psychological point of view, it has been suggested that the cognitive processing of art produces affective and often positive and pleasing aesthetic experiences. According to the information-processing stage model of aesthetic processing by Leder et al. (2004), the occurrence of aesthetic pleasure depends on a satisfactory cognitive understanding of the artwork. The better the understanding, the more the reduction of ambiguity, and the higher the probability of positive aesthetic emotion. When aesthetic experiences are often positive, it can be expected an increase in positive affect (Leder et al., 2004). Enduring predominance of diffuse positive affective states influences mood (Scherer, 2005), promotes health and learning. Consistently, some neurophysiological studies find that context information facilitates the processing of a work of art and increases positive emotions (Gerger and Leder, 2015; Mastandrea, 2015; Mastandrea and Umiltà, 2016). This is accompanied by greater neural activity in the medial orbitofrontal cortex (OFC) and ventromedial prefrontal cortex, regions strongly associated with the experience of reward and emotion processing (Kawabata and Zeki, 2004; Kirk et al., 2009).

On the other hand, various theories of emotion have been influential in describing the paradoxical enjoyment of negative emotions in art (Juslin, 2013; Sachs et al., 2015; Menninghaus et al., 2017). Several authors suggested that the psychological distance of the perceiver from what is depicted in the artwork—which comes from the individual’s awareness that the represented object or event is a cultural artifact—reduces the emotional impact of the eliciting object or event and allows the appraisal of the aesthetic qualities of the artwork. This “psychological distance” account underpins the difference between art-specific emotions and utilitarian emotions (Frijda, 1988; Scherer, 2005). Perceiving safety during art reception allows negative content of the artwork to be embraced. In this account, negative emotions such as sadness and sorrow are transformed in source of pleasure and empathetic responses to the emotional content of the artwork are allowed by the meta-emotional reappraisal (Menninghaus et al., 2017). Accordingly, art context influenced aesthetic judgment and emotional responses as measured by facial electromyography (EMG). Specifically, defining visual stimuli as artistic prompted participants to judge artworks depicting negative emotional content more positively, meaning “liked” more. In other words, there might be a general positive bias in the perception of art (Gerger et al., 2014).

The pleasurable effect of negative emotions in art reception has been extensively investigated in the field of music (Vuoskoski et al., 2012; Juslin, 2013; Kawakami et al., 2013; Taruffi and Koelsch, 2014; Sachs et al., 2015). According to the BRECVEMA model elaborated by Juslin (2013), enjoying sadness in music derives from the combination of two key mechanisms, i.e., emotional contagion and aesthetic judgment that generate mixed affective responses. While listening to sad music, one may experience the feeling of sadness through the mechanism of emotion contagion and appreciate the beauty of the piece by judging it aesthetically positive (Juslin, 2013). Some authors described the beneficial effects of music listening on the emotional health, reporting that listeners use music to enhance positive emotions and regulate negative emotions, affecting mood (Taruffi and Koelsch, 2014; Sakka and Juslin, 2018). Consistently, an influential model by Sachs et al. (2015) posits that pleasure in response to sad music is functional to restore homeostatic equilibrium that promotes optimal functioning. For instance, a person who is experiencing emotional distress and has an absorptive personality will find pleasure in listening to sad music because, being focused on the aesthetic experience of appreciating the beauty of music will disengage him/her from distress, promoting positive mood. This concept is supported by the fact that listening to sad music engages the same network of structures in the brain (i.e., the OFC, the nucleus accumbens, insula, and cingulate) that are known to be involved in processing other stimuli with homeostatic value, such as those associated with food, sex, and attachment (Berridge and Kringelbach, 2015; Sachs et al., 2015).

In line with the conceptual frameworks offered by music research, it may be hypothesized that pleasure in visual art reception relies upon (1) emotional contagion with the valence conveyed by the artwork; (2) appraising a negative emotional stimulus as a fictional rather than realistic; (3) regulating emotion accordingly; (4) enjoying aesthetic experience and performing aesthetic judgment. If aesthetically pleasing, such an experience can be defined rewarding. The dynamic interaction of these and other factors for producing pleasurable aesthetic experience has been broadly described in theories of aesthetic processing (e.g., Sachs et al., 2015; Menninghaus et al., 2017; Pelowski et al., 2017). Providing a comprehensive account of this complex process is out of the scope of this review; however, here we focus on how a part of these mechanisms—i.e., emotion contagion, emotion regulation, pleasure, and reward—find a common neural substrate in network of emotion processing and how coupling neuroimaging research with measurement of physiological states may be useful for demonstrating a link between aesthetic experience and promotion of well-being.

Neuroaesthetics is a relatively recent research field within cognitive neuroscience and refers to the study of neural correlates of aesthetic experience of beauty, particularly in visual art (Chatterjee and Vartanian, 2016). Using multimodal neuroimaging techniques, such as functional magnetic resonance (fMRI), magnetoencephalography (MEG), and electroencephalography (EEG), it has produced heterogeneous results. Most studies, however, converge in the consideration of the orbitofrontal cortex (OFC), and more generally, the core centers of emotional and reward-related responses as the putative correlates of the aesthetic experience of beauty (Kawabata and Zeki, 2004; Di Dio and Gallese, 2009; Ishizu and Zeki, 2013), hence supporting psychological studies that suggest that aesthetic experience is emotionally positive and rewarding (Leder et al., 2004). Using fMRI, it has been shown that rating the beauty of an artwork selectively engaged regions within the OFC irrespective of stimulus type (i.e., visual art, visual texture, music, mathematical formulae, moral judgment etc.) (Blood et al., 1999; Kawabata and Zeki, 2004; Tsukiura and Cabeza, 2011; Jacobs et al., 2012; Zeki et al., 2014). Moreover, metabolic activity in those areas increased linearly as a function of aesthetic, but not perceptual judgment of paintings (Ishizu and Zeki, 2013), indicating that aesthetic preference for paintings is mediated by activity within the reward-related network. Similarly, using MEG to record evoked potentials while viewing images of artworks and photographs, Cela-Conde et al. (2004) found that the left dorsolateral prefrontal cortex (DLPFC) responded more when participants judged the images as beautiful, than when they judged the images as not beautiful (Cela-Conde et al., 2004). Interestingly, Vartanian and Goel (2004) highlighted different neural patterns of activation for pleasant and unpleasant paintings. Specifically, they found that bilateral occipital gyri and left cingulate sulcus activated more in response to preferred stimuli, whereas activation in the right caudate nucleus decreased in response to decreasing preference ratings (Vartanian and Goel, 2004). As activity in the caudate nuclei have been found to decrease following a punishment feedback (Delgado et al., 2000), it may be suggested that deactivation of left caudate reflects a general pattern of reduced activation to less rewarding stimuli (Vartanian and Goel, 2004). In line with these findings, a recent study of Ishizu and Zeki (2017) showed that images rated as beautiful but evoking opposite emotions (i.e., joy vs. sorrow) modulated activity in OFC, but also activated areas that have been found to be involved in positive emotional states (i.e., controlling empathy toward other)—such as the temporoparietal junction (TPJ) and the supramarginal gyrus (SMG)—and negative emotional states (i.e., perception of social pain)—such as the inferior parietal lobule (IPL) and the middle frontal gyrus (MFG) (Ishizu and Zeki, 2017). Consistent with these findings, theories of embodied cognition suggested that emotions may be conveyed by the work of art through embodied simulation (Freedberg and Gallese, 2007; Azevedo and Tsakiris, 2017) or motor contagion (Gerger et al., 2018). In support of this, neuroimaging studies found the aesthetic judgment of human and nature content paintings to be modulated by the activation of a motor component. That is, cortical motor systems were activated including parietal and premotor areas (Di Dio et al., 2015). This suggests that dynamic artworks may engage motor systems *via* features that represent actions and emotions (Freedberg and Gallese, 2007).

Therefore, experiencing art is a self-rewarding activity, irrespective of the emotional content of the artwork. This finding is supported by previous research showing that an art context heightens positive response toward images with negative content (Gerger et al., 2014). Adopting a distanced perspective in art reception may produce positive emotional state and pleasure, irrespective of the emotional content of the artwork (Leder et al., 2004; Menninghaus et al., 2017). Moreover, it appears that art-specific emotions and utilitarian emotions found a common neural substrate in brain network involved in emotion processing and reward.

### Aesthetic Emotion and Well-Being: Which Relationship?

The studies reviewed so far suggest that the aesthetic processing of an artwork can elicit in the beholder affective states congruent with those evoked by the artwork itself (Freedberg and Gallese, 2007; Azevedo and Tsakiris, 2017; Ishizu and Zeki, 2017).

Critically, the positive or negative valence of the aesthetic emotion does not appear to be relevant in determining the reward value of the aesthetic experience. A portrait, a sculpture, or a piece of music conveying feelings of sadness may be rated as beautiful and produce a modulation onto OFC regions and the centers of reward-related responses similar to artworks conveying positive feelings, such as joy and pleasure. These results support the claim that adopting a psychological distance in art context allows the perceiver to embrace the negative content of the work of art and, by means of empathetic responses to the content of the artworks, provoking aesthetic pleasure (Menninghaus et al., 2017). According to Marković (2012), the aesthetic experience is an exceptional state of mind, which opposes everyday, pragmatic experience and “protects” the individual from the effects of oppressive reality (Marković, 2012). Given these considerations, it may be thought that the aesthetic emotion is distinctive of aesthetic appreciation, denoting an art-specific emotional response evolved from basic biologic emotions (Leder et al., 2004). As such, this self-rewarding nature of aesthetic experience may account for aesthetic appreciation’s promotion of health and well-being. Alternatively, it may be that experiencing positive aesthetic emotions is not only the outcome of a special empathetic state provoked by the artwork but may depend on the level of perceived ambiguity in the artwork itself. In *processing fluency theory of beauty*, the more fluently the perceiver can process an object, the more positive the aesthetic response (Reber et al., 2004). In other words, features that facilitate processing of a stimulus (e.g., objective stimulus properties and subjective previous experience with the stimulus) result in positive affective responses and more favorable judgments or preferences (Reber et al., 2004). In this view, positive valence of the aesthetic emotion is the product of the processing experience of the perceiver, aesthetic or not.

Therefore, aesthetic pleasure can depend, in turn, on satisfactory mastering the stimulus, emotional responses or both (Mastandrea et al., 2009; Chirumbolo et al., 2014). As reviewed above, theoretical frameworks explaining the paradox of enjoying negative emotions in art indicated that different key factors interact to produce a pleasurable response (Juslin, 2013; Menninghaus et al., 2017), as a function of restoring homeostatic balance (Sachs et al., 2015).

Intriguingly, the positive affective state derived from the aesthetic emotion, whatsoever origin it may have had, may have a common neural substrate in the reward-related brain circuitry.

Nevertheless, these different approaches to aesthetic evaluation may have different implications for a strategic use of art as tool for promoting well-being and health. Consistent with the fluency processing theory of beauty, representational paintings should be more effective than abstract paintings for enhancing learning processes within art-based education programs. Similarly, artwork high in comprehensibility should render healthcare settings or work environments more gratifying than less intelligible artwork. On the other hand, it is possible that experiencing an abstract modern painting in an art museum (i.e., an art context soliciting the adoption of a distanced perspective in the perception of art) can arouse a powerful aesthetic emotion. This could improve perceived well-being (Freedberg and Gallese, 2007; Gerger et al., 2014, 2018; Menninghaus et al., 2017).

Unfortunately, as far as we know, there are only a few studies that explore the neural correlates associated with cognitive- or affective-based accounts of the aesthetic experience and their relation to the use of art for promoting individual well-being. Furthermore, most empirical investigations of the relationship between art and well-being do not consider objective measures of stress, such as skin conductance, heart rate variability, or respiration rate. Further, any conclusion about a relationship between art appreciation and well-being is hampered by the use of quite different subjective measures of well-being, such as interviews and questionnaires. Nowadays, we know from the literature that the pleasure associated with aesthetic processing may be modulated by emotional responses of the beholder to the artwork, or may be function of the successful cognitive mastery of the aesthetic stimulus (Leder et al., 2004; Menninghaus et al., 2017; Gerger et al., 2018), or may be a function of a more complex model. Deeper understanding of the dynamic relationship between bottom-up stimulus properties and top-down cognitive appraisal on emotional experience during the aesthetic appreciation of an artwork might be useful to effective use of art-based tools for promoting individual health and well-being. Investigating the interplay between art and well-being must not omit consideration of the analysis of more objective psychophysiological measures of stress, such as autonomic responses. Future research should address the relationship between the emotional responses to aesthetic and non-aesthetic stimuli and measures of well-being, such as combining neural responses with autonomic indices of stress.

## Conclusion

Aesthetic experience, in many settings, may promote well-being. Neuroaesthetics research suggests that aesthetic pleasure is derived by the interaction between emotion processing that involves reward-related areas in the brain and top-down processes derived from the relationship of the beholder with the cultural artifact. The self-rewarding nature of aesthetic experience may influence the beholder’s affective state, possibly improving well-being. However, there still are many questions that future research should address to clarify the determinants of aesthetic pleasure and their relationship to health. First, the impact of aesthetic emotion on measured well-being has been assessed through subjective ratings using interviews or questionnaires, scarcely considering more objective indices recorded through psychophysiological measures. Moreover, it remains unclear whether proper use of art to improve well-being should emphasize the empathetic responses to the artwork or the possibility for the beholder to master the meaning of the artwork itself. Future research should consider these issues in developing art-based programs in healthcare and education.

## Author Contributions

SM conceived the idea, reviewed the literature, and wrote the draft of the manuscript. SF reviewed the literature and wrote the draft of the manuscript. VB collaborated to the idea with SM, reviewed the literature on educational applications and supervised the manuscript writing.

### Conflict of Interest Statement

The authors declare that the research was conducted in the absence of any commercial or financial relationships that could be construed as a potential conflict of interest.
